# Complex biology of constitutional ring chromosomes structure and (in)stability revealed by somatic cell reprogramming

**DOI:** 10.1038/s41598-021-83399-3

**Published:** 2021-02-22

**Authors:** T. V. Nikitina, A. A. Kashevarova, M. M. Gridina, M. E. Lopatkina, A. A. Khabarova, Yu. S. Yakovleva, A. G. Menzorov, Yu. A. Minina, I. E. Pristyazhnyuk, S. A. Vasilyev, D. A. Fedotov, O. L. Serov, I. N. Lebedev

**Affiliations:** 1 Research Institute of Medical Genetics, Tomsk National Research Medical Center, Ushaika Street 10, Tomsk, 634050 Russia; 2 Department of Molecular Mechanisms of Development, Institute of Cytology and Genetics SB RAS, Novosibirsk, 630090 Russia; 3 Department of Medical Genetics, Siberian State Medical University, Tomsk, 634050 Russia; 4 Department of Natural Sciences, Novosibirsk State University, Novosibirsk, 630090 Russia

**Keywords:** Genetics, Stem cells

## Abstract

Human ring chromosomes are often unstable during mitosis, and daughter cells can be partially or completely aneuploid. We studied the mitotic stability of four ring chromosomes, 8, 13, 18, and 22, in long-term cultures of skin fibroblasts and induced pluripotent stem cells (iPSCs) by GTG karyotyping and aCGH. Ring chromosome loss and secondary aberrations were observed in all fibroblast cultures except for r(18). We found monosomy, fragmentation, and translocation of indexed chromosomes. In iPSCs, aCGH revealed striking differences in mitotic stability both between iPSC lines with different rings and, in some cases, between cell lines with the same ring chromosome. We registered the spontaneous rescue of karyotype 46,XY,r(8) to 46,XY in all six iPSC lines through ring chromosome loss and intact homologue duplication with isoUPD(8)pat occurrence, as proven by SNP genotype distribution analysis. In iPSCs with other ring chromosomes, karyotype correction was not observed. Our results suggest that spontaneous correction of the karyotype with ring chromosomes in iPSCs is not universal and that pluripotency is compatible with a wide range of derivative karyotypes. We conclude that marked variability in the frequency of secondary rearrangements exists in both fibroblast and iPSC cultures, expanding the clinical significance of the constitutional ring chromosome.

## Introduction

Human non-supernumerary constitutional ring chromosomes are rare chromosome structural abnormalities (1:50,000 newborns)^1^ and can be found for all human chromosomes. Constitutional ring chromosomes are generally believed to be the result of de novo breakage of both end-segments of a chromosome during meiosis or early postzygotic mitosis. Other mechanisms include junction of the telomeric or subtelomeric regions without loss of genetic material^2,3^ and inverted duplication associated with a terminal deletion (inv dup del rearrangements), which may be stabilized through circularization^4–7^. Due to their circular structure, ring chromosomes may have problems in mitosis, which depends on sister chromatid exchange (SCE). Depending on the number and position of the crossovers, a ring chromosome can produce dicentric and interlocked rings and thus undergo anaphase lagging, non-disjunction, or fragmentation during cell division, resulting in the production of cells without a ring chromosome, with many ring chromosomes, nuclear projections and micronuclei^8^. Various ring derivatives may be generated, and daughter cells can be partially or completely aneuploid, thus having reduced proliferative activity or viability due to secondary rearrangements^3,9^.

The specific constitutional ring behaviour, mitotic instability, is a source of “dynamic tissue-specific mosaicism”, as described by McDermott et al*.*^10^ in 1977. The tissues of ring carriers are often mosaics due to the presence of ring chromosomes and their derivatives, which leads to phenotypic consequences^11^. The mitotic instability of ring chromosomes and the subsequent death of part of the cells may also explain the so-called “ring chromosome syndrome” that is observed in some patients, regardless of the chromosome involved, and is associated with growth retardation in the absence of serious malformations^12^. Different ring chromosomes have different levels of in vivo stability depending on their sizes and gene content^3^, but there is no unambiguous correlation between the size of a ring chromosome, the prevalence of dynamic mosaicism, and the severity of phenotypic manifestations in a patient^6^.

The occurrence of secondary rearrangements in carriers of ring chromosomes is probably a more common phenomenon than previously believed based on the results of metaphase cytogenetics^13–15^. The current methods of detailed genetics analysis of various tissues will allow more precise assessment of the real frequency of partial or complete aneuploidy resulting from secondary rearrangements and mosaicism of ring chromosomes^16,17^. As shown recently, the complex mitotic behaviour of ring chromosomes can lead to specific patterns of their segregation in different tissues^18–20^.

We propose the use of induced pluripotent stem cells (iPSCs) as an approach for studying the tissue-specific mitotic instability of ring chromosomes. Unlike differentiated cells, iPSCs have unlimited proliferative ability, which enables tracing of the mitotic behaviour of ring chromosomes in actively dividing cells over a significant number of cell divisions. Moreover, using iPSCs as analogues of embryonic stem cells (ESCs) makes it possible to simulate the segregation of ring chromosomes and their derivatives between daughter cells during early postzygotic mitotic cell division and in the inner cell mass (ICM), which affects the subsequent status of the ring chromosome in derivatives of different germ layers and tissues that are usually unavailable for cytogenetic analysis in patients with constitutional ring chromosomes.

Recently, cases of “compensatory” uniparental disomy (UPD) of chromosomes 17 and 13 in iPSCs were described; the authors assumed that mitotic inheritance of ring chromosomes in iPSCs is very unstable and that ring chromosomes are incompatible with pluripotency^21^. Cell-autonomous correction of ring chromosomes in human iPSCs has been proposed as a way to achieve “chromosome therapy” for large-scale chromosomal aberrations^21,22^.

The aim of this study was to determine the possibility of producing iPSC lines with ring chromosomes and to analyse the numerical and microstructural (in)stability of ring chromosomes in cultures of somatic cells (fibroblasts) and pluripotent cells. We reprogrammed skin fibroblasts from four patients with constitutional ring chromosomes 8, 13, 18, and 22; obtained iPSC lines; and found marked variability in the mitotic stability of ring chromosomes not only in iPSCs but also in fibroblasts. We observed the spontaneous correction of the iPSC karyotype with ring chromosome 8 to normal through the formation of chromosome 8 uniparental isodisomy (isoUPD(8)pat). iPSCs lines with stable rings can be differentiated into certain types of cells and used to create cellular models of the chromosomal diseases.

## Results

### Ring chromosome 8 (r(8))

**Figure 1:**
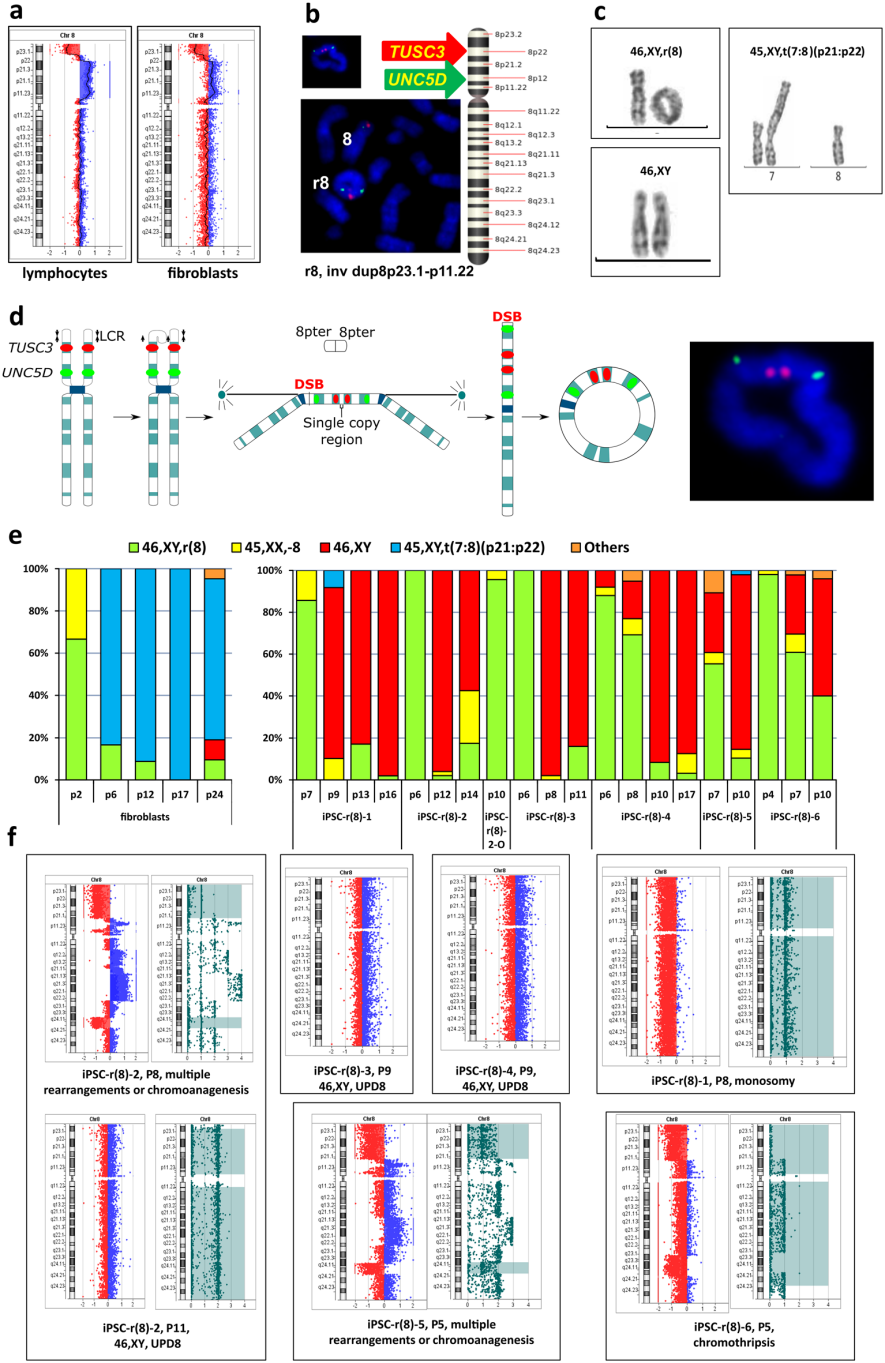
Results of GTG and molecular karyotyping of cell lines with r(8). (**a**) aCGH profiles of chromosome 8 in lymphocytes (left) showing a terminal deletion of 8p23.3-p23.1 and duplication at 8p23.1-p11.22 and fibroblasts (right) showing the complex structure of the short arm of chromosome 8 with del8p23.3-p23.1, del8p23.1, dup8p12-p11.23, and del8p11.22-p11.1 [CytoGenomics (v. 3.0.6.6) (https://www.agilent.com/en/download-agilent-cytogenomics-software)]. (**b**) FISH analysis showing r(8) morphology and confirming the inverted status of a duplication (invdup8p23.1-p11.22) and the approximate positions of the FISH probes on chromosome 8 (red: probe for the unique gene *TUSC3*; green: probe for the unique gene *UNC5D*) [ISIS software (v.5.5), MetaSystems (https://metasystems-international.com/en/products/isis/)]. (**c**) Examples of chromosome 8 pairs in fibroblasts and iPSCs with r(8) and its derivatives: translocation t(7:8) and disomy 8. (**d**) Possible mechanism of r(8) origin: exchange between low-copy repeats leading to the formation of dicentric chromosome and acentric fragment, containing two terminal regions of chromosome 8. This resulted in the deletion of the 8p23.3-p23.1 region. During cytokinesis, a chromatid break occurred in the 8p11.22 region, leading to the deletion of the 8p23.1-p11.22 region in one daughter cell and duplication of this region in another daughter cell. Then the chromosome was closed into the observed ring chromosome, which contains a duplication of the region 8p23.1-p11.22 and deletion of the region 8p23.3-p23.1. (**e**) Karyotype rates in fibroblasts (left) and iPSC lines (right) at different passages. (**f**) aCGH profiles of iPSC lines demonstrating iPSC-r(8)-2 at P8 with signs of multiple rearrangements or chromoanagenesis and at P11 with two copies of chromosome 8 (UPD8), iPSC-r(8)-1 at P8 with monosomy 8, iPSC-r(8)-3 at P9 with UPD8, iPSC-r(8)-4 at P9 with UPD8, iPSC-r(8)-5 at P5 with signs of multiple rearrangements or chromoanagenesis, and iPSC-r(8)-6 at P5 with loss of most of the genetic content of chromosome 8 or chromothripsis [CytoGenomics (v. 5.0.2.5) (https://www.agilent.com/en/download-agilent-cytogenomics-software)].

We obtained lymphocytes and fibroblasts from a patient with r(8) and Birk-Barel syndrome^23^, features of patients are briefly described in Table 1. Molecular karyotyping of lymphocytes revealed that the ring chromosome originated from a terminal deletion at 8p23.3-p23.1 (7.888 Mb) with a large (27.120 Mb) duplication at 8p23.1-p11.22 (Fig. 1a), which inverted status was confirmed by fluorescence in situ hybridization (FISH) (Fig. 1b). It is suggested that this ring chromosome 8 arose as a result of exchange between low-copy repeats^24^, the mechanism, which produce the normal copy region between the regions of deletion and duplication, as it was observed in our case (Fig. 1d). DNA from fibroblasts at passage 2 (P2) showed a more complex structure of the short arm of chromosome 8 with three deletions and one duplication (del8p23.3-p23.1 (7.888 Mb), del8p23.1 (3.749 Mb), del8p11.22-p11.1 (3.738 Mb), and dup8p12-p11.23 (3.244 Mb)) (Fig. 1a). GTG analysis revealed that 67% fibroblasts had r(8) and 33% fibroblasts had monosomy 8 at P2 (Fig. 1e), and interphase FISH with the centromeric probe D8Z2 showed monosomy 8 in 258 cells (51.7%) and disomy 8 in 241 cells (48.3%) (Supplementary Fig. S1). Unexpectedly, later cells with t(7;8) arose in the fibroblast culture (Fig. 1c); these cells had a proliferative advantage with more than 80% of the fibroblast at P6, P12, P17 and P24 (Fig. 1e).

**Table 1:** Cytogenetic and molecular findings in patients with ring chromosomes.

Ring chr	Absolute size, Mb	Age/sex	Main phenotype features	Genome imbalance
8	147.6	1.4/male	Neuropsychological developmental delay; muscle hypotonia; microcephaly; anomalies of the face, hands, and feet	invdup8p23.1-p11.22, del8p23.3-p23.1
13	113.6	17/male	Neuropsychic and speech delay, anxiety, macrocephaly, microorchidism, multiple anomalies of the internal organs	del13q34
18	63.2	3/male	Delayed psychomotor and speech development, bilateral ptosis of the eyelids, tooth abnormalities, hypermobility of the joints and violations of skin pigmentation	del18p11.32-p11.21, del18q23
22	49.2	6/female	Delayed psychomotor and speech development, hyperactivity, sleep disorders, multiple developmental anomalies, Phelan–McDermid syndrome	dup22q13.32, del22q13.32-q13.33

We generated iPSCs from patient’s fibroblasts at P2 using episomal vectors^25^. The karyotypes of the six iPSC lines studied were similar to those of the initial fibroblasts in the early passages (90% of the cells had karyotype 46,XY,r(8) at P4-6), but after P8-10, cells with the normal 46,XY karyotype prevailed in all six cell lines (Fig. 1e). Interestingly, metaphases with t(7;8) were also sporadically found in two cell lines: iPSC-r(8)-1 at P9 and iPSC-r(8)-5 at P10.

SNP-array analysis revealed signs of multiple rearrangements of chromosome 8 in the cell line iPSC-r(8)-2 at P8 and the “normal” karyotype, 46,XY, at P11 with isodisomy of chromosome 8: a normal copy number of chromosome 8 with loss of heterozygosity (LOH) along nearly all chromosome (Fig. 1f). For SNP-genotyping of the lymphocytes of the father and mother of the patient and iPSCs from his fibroblasts, a total of 3499 SNP markers on chromosome 8 were analysed. A total of 210 SNPs were informative, which indicated that both homologues of chromosome 8 in iPSC-r(8)-2 at P11 were of paternal origin. The genotype distribution provided evidence of isoUPD(8)pat.

Other iPSC lines also showed sequential stages of r(8) elimination: UPD(8) was found in the iPSC-r(8)-3 and iPSC-r(8)-4 cell lines at P9, and monosomy 8 was found in the iPSC-r(8)-1 cell line at P8. Two other cell lines, analysed at P5, demonstrated evidence of multiple rearrangements (iPSC-r(8)-5) and loss of most of the chromosome 8 genetic content (iPSC-r(8)-6) (Fig. 1f). Taken together, the GTG karyotyping and SNP array data suggest that in iPSC lines with r(8), self-correction of the karyotype to normal occurs via loss of the ring and uniparental isodisomy of an intact homologue of chromosome 8.

Only one iPSC-r(8)-2 subclone, iPSC-r(8)-2-O, retained r(8) without karyotype correction. This subclone showed degeneration of colony morphology, decreases in the expression of pluripotency markers, and subsequent differentiation at P14 (Supplementary Fig. S1). The remaining cell lines had ESC-like morphology of colonies, expression of markers of pluripotency (Supplementary Fig. S1) and differentiated into cell derivatives of all three germ layers, fully consistent with iPSC status.

### Ring chromosome 13 (r(13))

**Figure 2:**
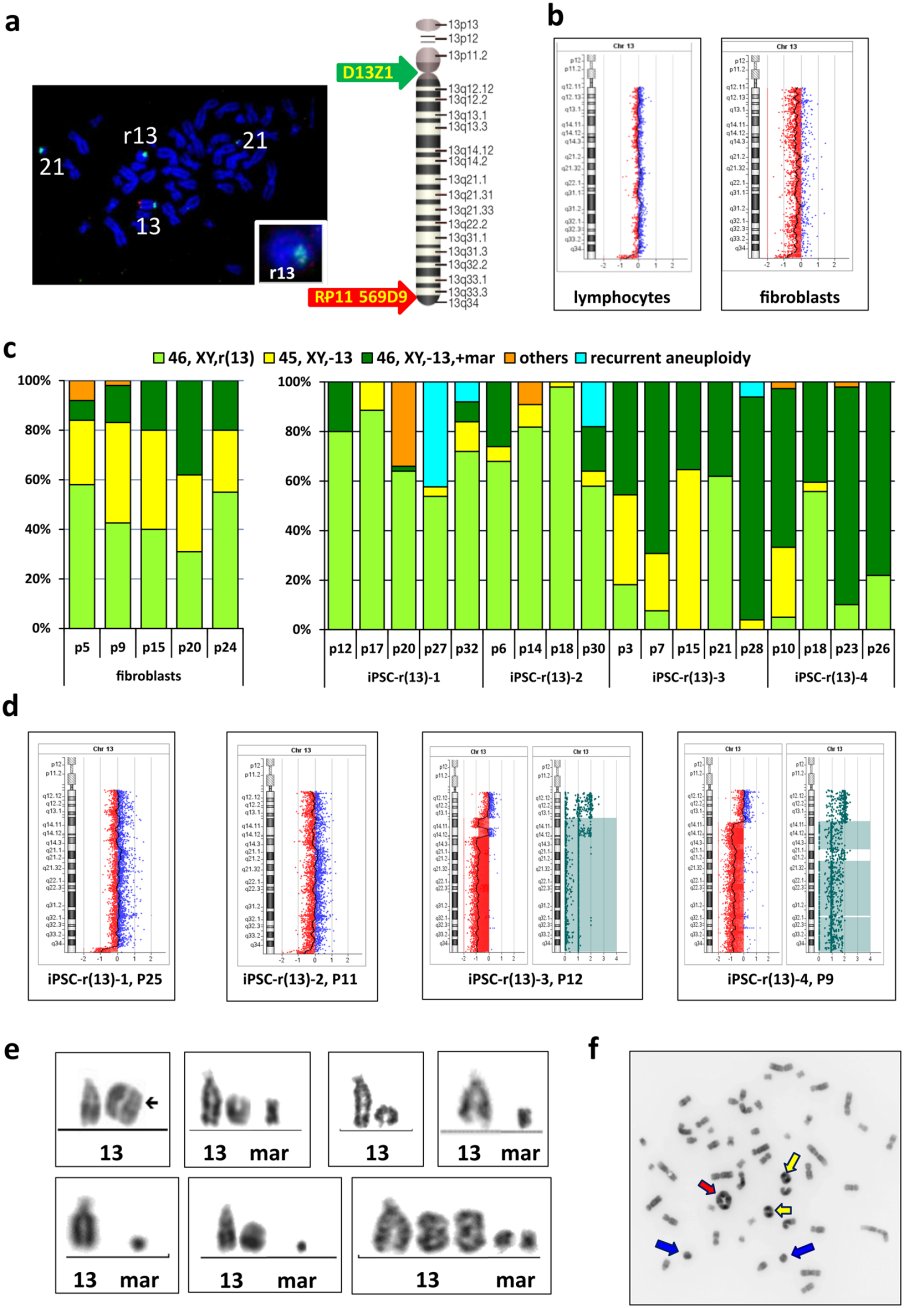
Results of GTG and molecular karyotyping of cell lines with r(13). (**a**) FISH analysis showing r(13) morphology (green: centromeric 13/21 probe D13Z1; red: probe RP11-569D9 on the distal part of the long arm of chromosome 13 (13q34), located in the deleted region) [ISIS software (v.5.5), MetaSystems (https://metasystems-international.com/en/products/isis/)]. (**b**) aCGH profiles of chromosome 13 in lymphocytes (left) showing two deletions at 13q34 and fibroblasts (right) with signs of monosomy 13 [CytoGenomics (v. 3.0.6.6) (https://www.agilent.com/en/download-agilent-cytogenomics-software)]. (**c**) Karyotype rates in fibroblasts (left) and iPSC lines (right) at different passages. (**d**) aCGH profiles of iPSC lines demonstrating iPSC-r(13)-1 at P25 with amplification of 13q31.3 (26 kb) and a terminal 13q34 deletion (2.53 Mb); iPSC-r(13)-2 at P11 with a 13q34 terminal deletion (1.987 Mb); iPSC-r(13)-3 at P12 with 5 large deletions covering most of the long arm and the normal copy region of 5.333 Mb surrounded by deleted regions within the LOH region of 80.116 Mb; and iPSC-r(13)-4 at P9 with multiple deletions on chromosome 13 and three LOH regions of 16.692 Mb, 32.472 Mb and 20.627 Mb, respectively [CytoGenomics (v. 3.0.6.6) (https://www.agilent.com/en/download-agilent-cytogenomics-software)]. (**e**) Examples of chromosome 13 pairs in fibroblasts and iPSCs with r(13) or derivative marker chromosomes. (**f**) Example of metaphase spread with amplification of r(13); red arrow: tetracentric ring, yellow arrows: dicentric rings, blue arrows: monocentric rings.

We obtained lymphocytes and fibroblasts from a patient with r(13), features of patients are briefly described in Table 1. Chromosome analysis revealed karyotype 46,XY,r(13)(p13q34)[18] in lymphocytes, confirmed by FISH (Fig. 2a), and a more complex karyotype in cultured skin fibroblasts: 46,XY,r(13)/46,XY,-13,+mar/45,XY,-13. The ratio of cells with these karyotypes was mostly stable at different passages, with a low prevalence of 46,XY,r(13) metaphases (Fig. 2c).

Molecular karyotyping of lymphocytes revealed two deletions at 13q34 (223 kb and 1.16 Mb) on chromosome 13 (Fig. 2b), but in fibroblast culture at P2, array-based comparative genomic hybridization (aCGH) recorded monosomy 13 in addition to a terminal deletion at 13q34 (2.099 Mb) (Fig. 2b).

Four iPSC lines with r(13), derived from patient’s fibroblasts^26^ using lentiviral vectors, showed a variety of karyotypes. Two cell lines, iPSC-r(13)-1 and iPSC-r(13)-2, consisted predominantly of cells with r(13), cells with monosomy 13 or with a marker chromosome (13 derivative) were minor classes. The other two cell lines, iPSC-r(13)-3 and iPSC-r(13)-4, showed pronounced variability in the frequencies of cells with different karyotypes, and on average, the major sub-population was composed of cells with a marker chromosome or even with monosomy 13 (Fig. 2c).

Molecular karyotyping of the cell lines iPSC-r(13)-1, iPSC-r(13)-2, iPSC-r(13)-3, and iPSC-r(13)-4 was performed at P25, P11, P12, and P9, respectively (Fig. 2d). The cell line iPSC-r(13)-1 showed amplification of 13q31.3 (26 kb) affecting a single gene, *GPC5*, that has not been described in fibroblasts; a terminal 13q34 deletion (2.53 Mb); and trisomy of chromosome 17. In the iPSC-r(13)-2 cell line, only a 13q34 terminal deletion (1.987 Mb) was identified. One of the most puzzling profiles of chromosome 13 was obtained in the cell line iPSC-r(13)-3, which exhibited five large deletions covering most of the long arm and a copy-neutral region of 5.333 Mb surrounded by the deleted regions. Surprisingly, this copy-neutral region was located within the LOH region of chromosome 13 (80.116 Mb) (Fig. 2d). The cell line iPSC-r(13)-4 also revealed multiple deletions on chromosome 13 as well as three LOH regions of 16.692 Mb, 32.472 Mb, and 20.627 Mb, respectively. Thus, two cytogenetically stable cell lines, iPSC-r(13)-1 and iPSC-r(13)-2, retained the terminal deletion similar to that in the initial ring chromosome, but two “unstable” cell lines revealed significant losses of the chromosome 13 genetic content (Supplementary Fig. S2). This outcome may have been caused by the coexistence of cell sub-populations with various lengths of deletions, especially as cytogenetic analysis revealed a wide range of marker chromosome variants of different sizes in these iPSC lines (Fig. 2e). The presence of the copy-neutral region surrounded by the deleted regions in iPSC-r(13)-3 may be a consequence of chromothripsis. However, the LOH in this area may indicate the probability of repair of the normal copy number due to recombination with the intact chromosome 13 homologue. We also found sporadic numerical amplifications of r(13) (Fig. 2f).

Despite the fact that the cell lines iPSC-r(13)-3 and iPSC-r(13)-4 were functionally similar to monosomy 13, they retained the normal ESC-like morphology of colonies and all the features of iPSCs, including the expression of pluripotency markers and the ability to differentiate into cell derivatives of all three germ layers in the embryoid bodies (Supplementary Fig. S2). At late passages (near P25), three of four cell lines with r(13) acquired trisomy of chromosome 12 or 17, each of which is a recurrent form of chromosomal aneuploidy in human pluripotent stem cell cultures^27–29^.

Thus, we found that in iPSC lines with r(13), both loss and fragmentation of the ring chromosome can occur; in addition, we found that pluripotency is compatible with a wide range of derivative karyotypes. Noticeable differences were found between the cell lines in the stability of r(13), which raises a question: why do isogenic iPSC lines with r(13) differ so much in their levels of mitotic instability?

### Ring chromosome 18 (r(18))

**Figure 3:**
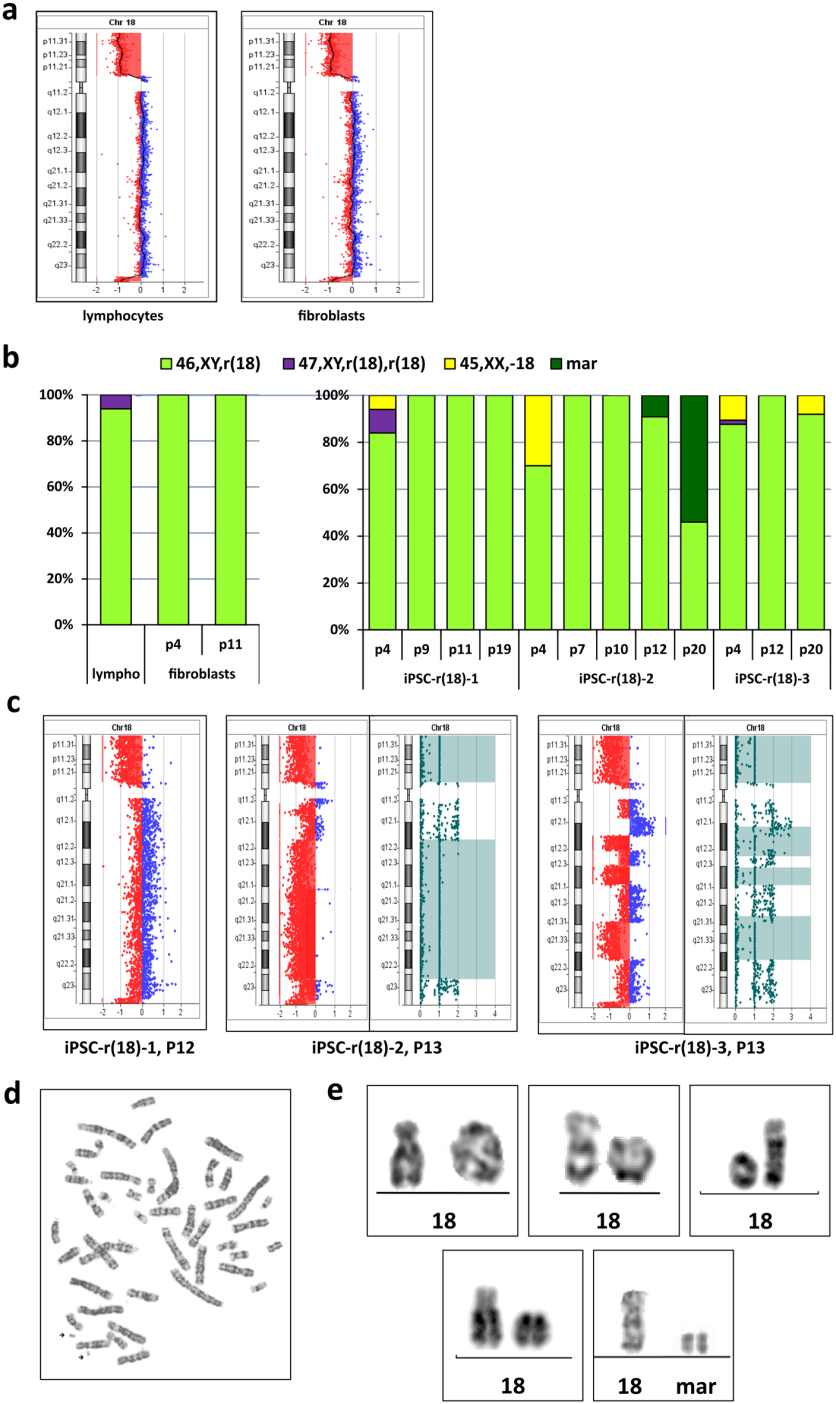
Results of GTG and molecular karyotyping of cell lines with r(18). (**a**) aCGH profiles of chromosome 18 showing a terminal deletion 1.238 Mb at 18q23 and a large (13.52 Mb) deletion at 18p11.32p11 in lymphocytes (left) and fibroblasts (right) [CytoGenomics (v. 3.0.6.6) (https://www.agilent.com/en/download-agilent-cytogenomics-software)]. (**b**) Karyotype rates in lymphocytes and fibroblasts (left) and iPSC lines (right) at different passages. (**c**) aCGH profiles of chromosome 18 in 3 iPSC lines: iPSC-r(18)-1 with a stable ring structure, iPSC-r(18)-2 with signs of multiple rearrangements or chromothripsis with the absence of most genetic content, and iPSC-r(18)-3 with signs of multiple rearrangements or chromothripsis [CytoGenomics (v. 5.0.2.5) (https://www.agilent.com/en/download-agilent-cytogenomics-software)]. (**d**) Example of metaphase spread with two marker chromosomes in line iPSC-r(18)-2. (**e**) Images of chromosome 18 pairs in fibroblasts and iPSCs with r(18) and its derivatives.

Lymphocytes and fibroblasts were obtained from a patient with r(18), features of patients are described in Table 1. Microarray analysis revealed two deletions on chromosome 18: a small (1.238 Mb) deletion of the terminal part of the long arm at 18q23 and a large (13.52 Mb) deletion that affected almost the entire short arm at 18p11.32p11.21 (Fig. 3a). The formed ring chromosome was stable: the lymphocyte karyotype was 46,XY,r(18)(p11.1q23)[47]/46,XY,der(18)r(18;18)(p11.1q23;q23p11.1)[3]. In fibroblasts, 100% of metaphases had karyotype 46,XY,r(18) at P4 and P11 (Fig. 3b). The deletions determined by aCGH were similar in lymphocytes and fibroblasts (Fig. 3a).

We generated iPSCs from patient’s fibroblasts at P3 using episomal vectors^30^. Three iPSC lines with r(18) were characterized by relatively high karyotype stability. At an early passage (P4), there was a small proportion of metaphases with monosomy 18 (6%, 30%, and 11% in cell lines iPSC-r(18)-1, iPSC-r(18)-2, and iPSC-r(18)-3, respectively), but after P7, almost 100% of the cells had karyotype 46,XY,r(18) (Fig. 3b). However, the mitotically stable ring chromosome was preserved until up to the 20^th^ passage only in cell line iPSC-r(18)-1, for which aCGH at P12 also revealed the absence of any new rearrangements (Fig. 3c). In iPSC-r(18)-2 at P13, aCGH revealed the loss of most of the chromosome 18 genetic material, although metaphase analysis at P12 found a marker chromosome—derivative 18—in only 10% of the metaphases. As expected, at P20, 54% of the cells in this cell line had the karyotypes 46,XY,-18,+mar or 47,XY,-18,+mar,+mar (Fig. 3d), indicating the predominance of the process of r(18) fragmentation at later passages (Fig. 3b,c). In iPSC-r(18)-3, GTG analysis revealed 100% of the cells with the ring at P12; 92% with the ring and 8% with monosomy 18 at P20 (Fig. 3b). Nevertheless, microarray analysis at P13 detected the presence of multiple deletions interspersed with copy-neutral areas and even duplication (Fig. 3c). The different GTG and microarray data may be explained by methodical differences: metaphase analysis registers mainly actively proliferating cells, whereas microarray analysis displays the quantitative ratio of cell sub-populations in the total DNA pool. Most likely, in iPSC-r(18)-3, a considerable proportion of cells underwent multiple rearrangements. Variants of ring chromosomes and markers, registered in these cell lines, are in Fig. 3e and Supplementary Fig. S3. Despite the difference in stability of the ring chromosome, all these cell lines had normal iPSC characteristics, including morphology of colonies and expression of pluripotency markers (Supplementary Fig. S3).

### Ring chromosome 22 (r(22))

**Figure 4:**
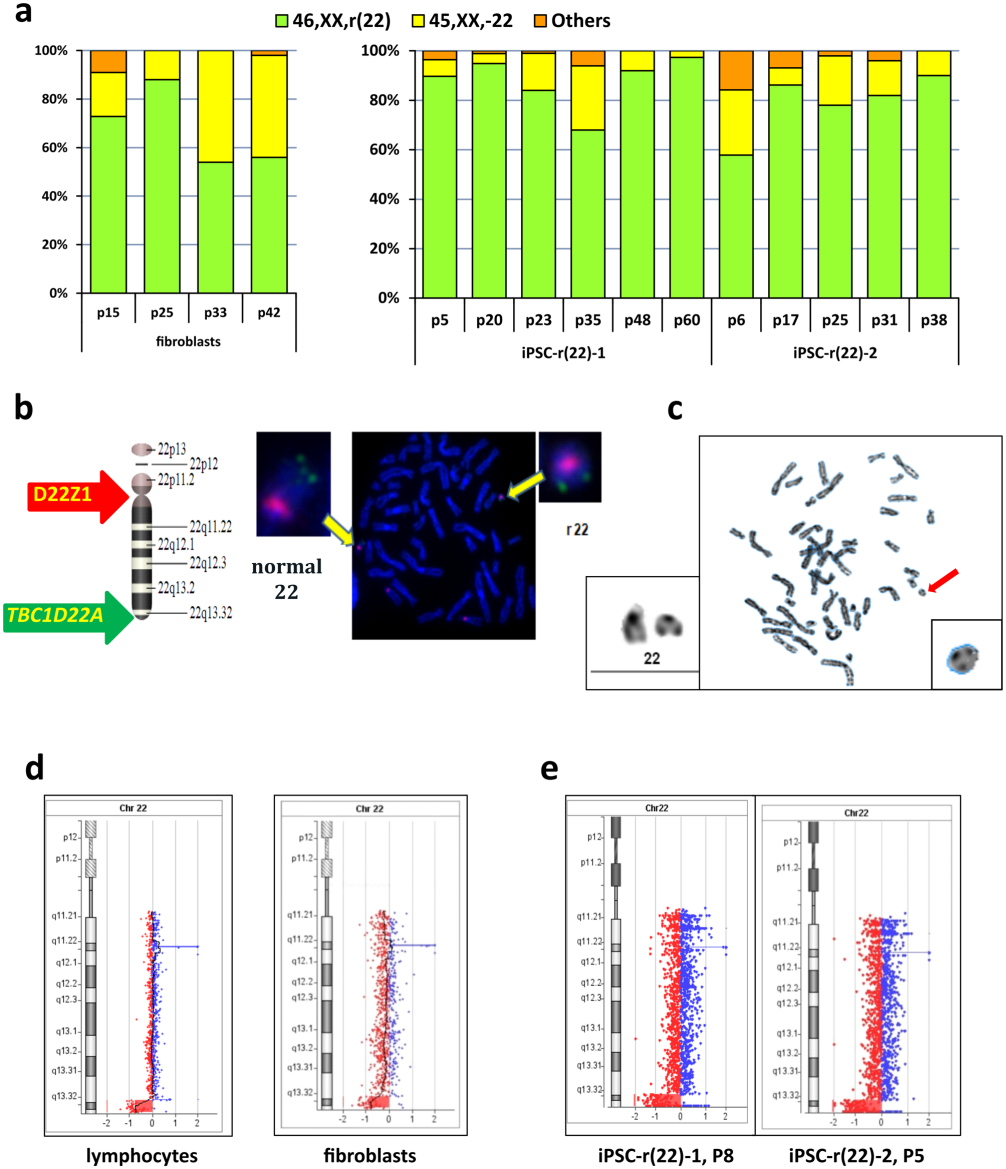
Results of GTG and molecular karyotyping of cell lines with r(22). (**a**) Karyotype rates in fibroblasts (left) and iPSC lines (right) at different passages. (**b**) FISH analysis showing the r(22) morphology and approximate positions of the FISH probes on chromosome 22 (red: centromeric probe 14/22, green: unique gene *TBC1D22A* (22q13.33), located outside the deleted region) [ISIS software (v.5.5), MetaSystems (https://metasystems-international.com/en/products/isis/)]. (**c**) Example of metaphase spread with r(22). (**d**) aCGH profile of chromosome 22 with a terminal deletion of 2.024 Mb at 22q13.32-q13.33 in lymphocytes (left) and fibroblasts (right) [CytoGenomics (v. 3.0.6.6) (https://www.agilent.com/en/download-agilent-cytogenomics-software)]. (**e**) aCGH profile of chromosome 22 in the two iPSC lines iPSC-r(22)-1 and iPSC-r(22)-2 with a stable ring structure [CytoGenomics (v. 5.0.2.5) (https://www.agilent.com/en/download-agilent-cytogenomics-software)].

We obtained lymphocytes and fibroblasts from a Phelan–McDermid syndrome patient with r(22)^31^, features of patients are briefly described in Table 1. Metaphase analysis of the G-banded chromosomes from peripheral blood lymphocytes revealed 46,XX,r(22)/45,XX,-22 mosaicism with 8% monosomic cells. The skin fibroblast culture showed high-grade dynamic mosaicism of cells with and without r(22): from P15 to P42, the percentage of 46,XX,r(22) metaphases decreased from 73 to 56%, and the percentage of metaphases with monosomy 22 increased from 18 to 42%, respectively (Fig. 4a). Morphology of r(22) is shown in Fig. 4b and c, and in Supplementary Fig. S4. In lymphocytes, aCGH identified a small (180 kb) microduplication at 22q13.32 and a subtelomeric microdeletion 2.024 Mb in size at 22q13.32-q13.33, and a similar microdeletion was observed in skin fibroblasts from the patient (Fig. 4d). At the microduplication region of 22q13.32, aCGH demonstrated a shift in the chromosomal profile towards duplication; however, this shift did not reach the significance level due to the high variance of the fluorescence intensity among the chromosomes.

Only two iPSC lines^32^ were obtained from the patient’s fibroblasts using lentiviral vectors, and in both, metaphase analysis showed a stable prevalence of cells with r(22): karyotype 46,XX,r(22) was found in 68–97% of the metaphases in iPSC-r(22)-1 from P5 to P60 and 58–90% of the metaphases in iPSC-r(22)-2 from P6 to P38 (Fig. 4a). The remaining cells mainly had monosomy 22. Microarray analysis of the cell lines iPSC-r(22)-1 (P8) and iPSC-r(22)-2 (P5) showed only microdeletion of the 22q13.32-q13.33 region, which is in accordance with that detected in lymphocytes and fibroblasts (Fig. 4e). We found a rather stable structure of r(22) during iPSC culture without any fragmentation at the sub-microscopic level. Thus, iPSCs with r(22) have a relatively stable karyotype, cells with r(22) persist as a modal sub-population for dozens of passages, and the ring structure remains virtually invariable. This variant of r(22) is even more stable in iPSCs than in cultured fibroblasts.

## Discussion

In this study, we examined the numerical and microstructural stability of ring chromosomes 8, 13, 18, and 22 in fibroblasts and fibroblast-derived iPSCs.

In the fibroblast cell cultures, the mitotic stability of the ring chromosomes differed in all cases:

r(18) was stably maintained, and 100% of metaphases at P4 and P11 had karyotype 46,XY,r(18);

r(22) showed high-grade dynamic mosaicism of the cells with and without r(22), and the rate of metaphases with monosomy increased from 18 to 42% in long-term culture up to P42;

r(13) produced a complex karyotype with ring chromosome loss or fragmentation (46,XY,r(13)/46,XY,-13,+mar/45,XY,-13), and the ratio of the cells with these karyotypes was quite stable at different passages with a low prevalence of 46,XY,r(13) metaphases; and

r(8) was found in 67% of fibroblasts (with the other 33% of cells exhibiting monosomy) at P2, but later a clone of the cells with t(7;8) arose. This rearrangement seemed to reduce the problem of ring chromosome passage through mitosis, and such cells had a proliferative advantage (more than 80% of the metaphases at P6, P12, P17 and P24). Thus, different ring chromosomes showed various patterns of "mitotic behaviour" in fibroblasts in vitro.

**Figure 5:**
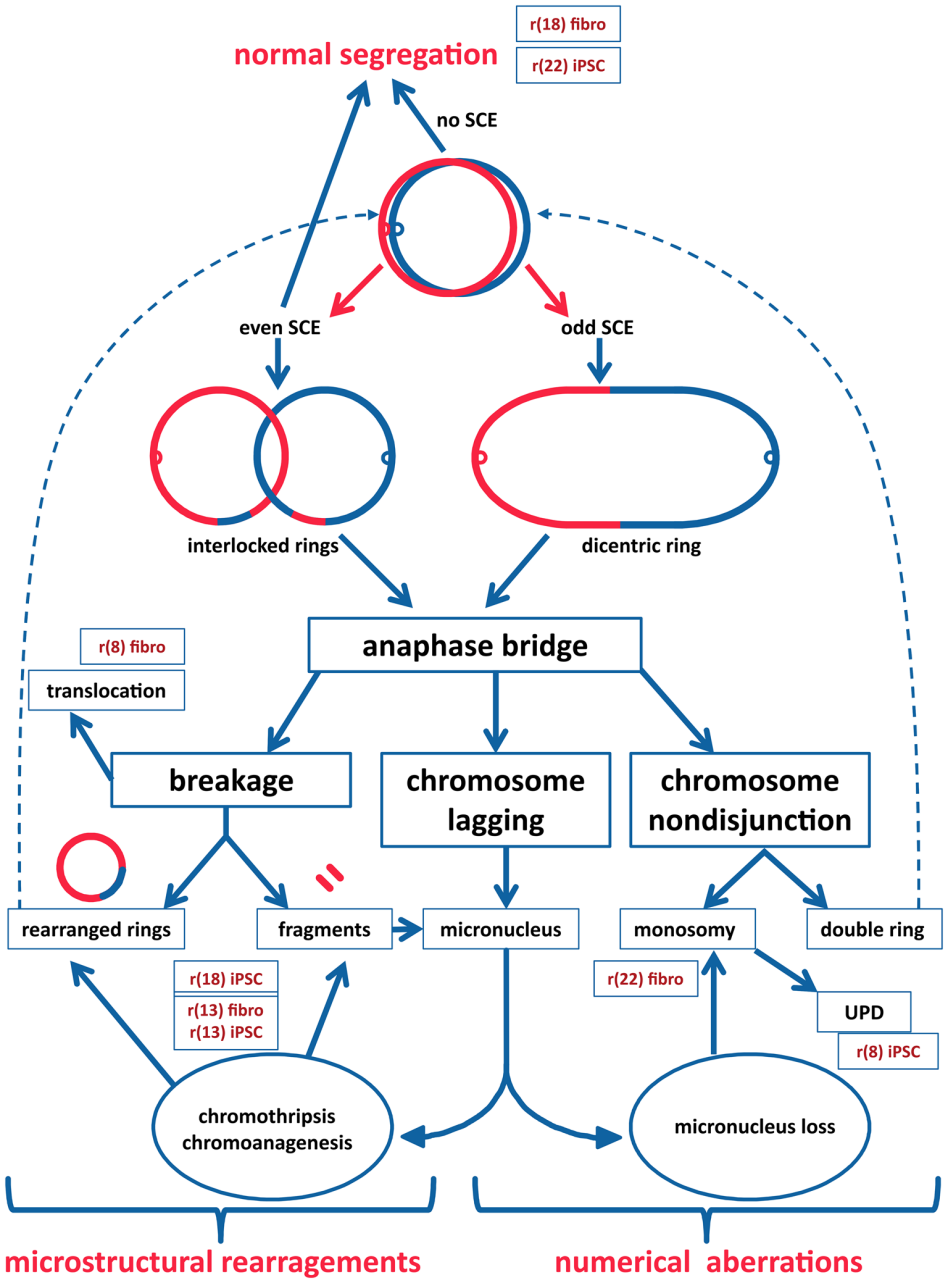
Hypothetical routes of ring chromosome mitotic instability. Possible mechanisms of the ring “behaviour” and formation of various ring derivatives found in this study for ring chromosomes 8, 13, 18, 22 in fibroblasts and iPSC cultures. If a ring chromosome replicates without SCE (no SCE) in prophase, the ring chromatids separate equally at anaphase, resulting in normal segregation to the daughter cells. One SCE (or an odd number of SCEs) causes the two daughter chromatids to form a dicentric double-sized ring. Two SCEs (or an even number of SCEs) would result in either normal segregation or the formation of interlocked rings. Dicentric rings and interlocked rings can form anaphase bridges at cell division with subsequent possible asymmetric breakage leading to unbalanced products or chromosome non-disjunction, resulting in the production of cells without a ring/with multiple rings. When a ring chromosome undergoes anaphase lagging, a micronucleus can form with either subsequent loss (resulting in monosomy) or reunion and chromothripsis (resulting in multiple rearrangements). As a result, numerical aberrations, microstructural rearrangements or normal segregation can be found. Obviously, combinations of different events can occur in a single cell line (and even in a single cell), so we present the names of the cell lines at the positions where the effect is observed more frequently. Dashed line arrows: passage to the next cell cycle.

Due to the circular structure, ring chromosomes encounter problems during mitosis, resulting in mitotic instability and abnormal segregation^8,33^. The fate of ring chromosomes in the cell cycle depends on SCE; the number and position of the crossovers affect the transmission of a ring to daughter cells^33,34^. When SCE occurs, the ring chromosome can form dicentric or interlocked rings with anaphase bridges during cell division and subsequently undergo anaphase lagging, non-disjunction, or breakage, resulting in cells without a ring, with multiple rings, or micronuclei; various ring derivatives may be produced, such as rings of different sizes or fragmentation of the rings (Fig. 5). Due to secondary rearrangements, daughter cells can be partially or completely aneuploid and have reduced proliferative activity or viability. The tissues of ring carriers are often mosaic due to the presence of ring chromosomes and their derivatives. Such dynamic mosaicism is hard to detect due to obvious limitations in obtaining different tissues for cytogenetic analysis. This limitation can be overcome by modelling ring chromosome instability in iPSCs.

In pluripotent cells, we found even more striking differences in mitotic stability both between iPSC lines with different rings and, in some cases, between different cell lines with the same ring chromosome. The iPSC lines with r(22) were the most stable: the 46,XX,r(22) karyotype was found in 97% and 90% of metaphases in the iPSC-r(22)-1 and iPSC-r(22)-2 lines at P60 and P38, respectively, and the structure of the ring remained almost invariable. Among iPSCs with r(18), only one of three cell lines remained stable until P20; aCGH of the other two cell lines at P13 showed loss of a significant portion of the chromosome 18 genetic content, which indicates that many cells underwent multiple rearrangements. r(13) was found to have the most interlineal variability in iPSCs. Two relatively stable cell lines, iPSC-r(13)-1 and iPSC-r(13)-2, retained a terminal deletion similar to that of the initial ring chromosome and had r(13) in most metaphases, but two “unstable” lines, iPSC-r(13)-3 and iPSC-r(13)-4, had significant losses of chromosome 13 genetic content. The reason may be coexistence of cells with various lengths of deletions as a result of fragmentation, as evidenced by a wide range of marker chromosome variants of different sizes revealed by cytogenetic analysis of these iPSC lines (Fig. 2e). Another possible explanation could be chromothripsis, when the lagging chromosome encapsulated within a micronucleus and then reincorporated into the nucleus of one of the daughter cells with loss of some DNA material. As a result, complex rearrangements occur simultaneously with copy-number changes^35,36^. However, in the iPSC-r(13)-3 cell line, we found a copy-neutral region surrounded by deleted regions within the LOH area, which indicates that repair of the normal copy number probably occurred through recombination with the intact homologue of chromosome 13, as chromothripsis must result in retained heterozygosity in copy-neutral rearranged regions^37^.

Therefore, two of four iPSC lines with r(13) and one of three with r(18) were much more stable in long-term culture than the other cell lines. The reason for this striking difference in the mitotic behaviour of ring chromosomes among isogenic cell lines remains unclear.

The karyotypes of the six iPSC lines with r(8) at the early passages were similar to those of the progenitor fibroblasts (approximately 90% of the cells had the 46,XY,r(8) karyotype), but after P8-10, cells with the 46,XY karyotype prevailed in all six cell lines (Fig. 1e). The results of microarray analysis and genotype distribution provided evidence of isoUPD(8)pat (Fig. 1f).

The detection of cells with normal karyotype among iPSCs could be a consequence of the presence of such cells in the initial cell population due to undetected low-level mosaicism^38,39^. However, the fact that all iPSC lines had a ring chromosome at early passages suggests that the cells with r(8) were reprogrammed and that the karyotype was normalized in iPSCs. We considered two possible mechanisms to explain the spontaneous karyotype normalization in the iPSC culture. The first mechanism is anaphase lagging leading to loss of the ring in daughter cells followed by monosomy correction via duplication of the remaining intact homologue with uniparental isodisomy (isoUPD)^40^. The second is ring opening due to a double-strand break (DSB)^41^ followed by homology-based repair or somatic recombination^42^ with the wild-type homologue accompanied by the formation of a stable linear structure. Both situations have been described in vivo and in vitro^41,43^. The fact that we found an LOH region along almost the entire length of chromosome 8 supported the first mechanism. SNP genotyping of the father, mother, and iPSCs confirmed that both homologues of chromosome 8 were of isodisomic paternal origin.

iPSCs are similar to ESCs in their properties and simulate early stage of the embryonic development, such as inner cell mass (ICM). We found normalization of the karyotype in iPSC lines with r(8) through the mechanism of “compensatory” uniparental disomy (UPD) and translocation t(7:8) in fibroblast culture; however, cells with such karyotypes were not found in the lymphocytes of our patient. One of the reasons for the differences observed in vivo (in patients) and in vitro (in cell cultures) is the size of the cell population. The number of cells in culture is limited and it also pass through the bottleneck when culture is established, that increase both the selective pressure of culture conditions and the likelihood of the mutation detecting, especially if mutation provides a proliferative advantage. In the organism consisting of billions of cells, mutational events, even if they do occur, will probably not be detected. For the normalization of the karyotype to be registered, it must occur very early in embryogenesis, and such cells must constitute a significant proportion of ICM cells. Further, depending on the tissue distribution of such cells, they may or may not be detected when examining the patient's tissues available for analysis.

According to published data, three out of nine previously reported patients with non-supernumerary r(8) were mosaic for euploid cells^43–45^. Unlike dynamic mosaicism, which constantly arises due to mitotic instability of the rings, such true mosaicism has other mechanism of formation.

The origin of euploid cells in patients^44,45^ is unclear, but in both, the normal cells were only found in fibroblasts. In study Gradek et al*.*^43^, patient with 46,XY,r(8)/46,XY mosaicism gives an example of rescue of monosomy 8 by chromosome duplication, resulting “compensatory” UPD (the mechanism similar with iPSC events in our study). The rate of the lymphocytes with a normal karyotype increased with the age of the patient (0%, 3%, and 23% at 9, 12 and 19 years, respectively), and 36% fibroblasts, obtained at 12 years, were euploid. A similar phenomenon of “compensatory” UPD was seen in a patient with r(21), where the 46,XX karyotype was present in 0.5% lymphocytes from the age of one month and in 97.3% lymphocytes from the age of 30 months^40^. While the lymphocyte karyotype was almost normalized, in fibroblasts at either age normal karyotype was not found at all. We suppose this is probably due to the initial inter-tissue distribution of mosaic cells in the early stages of embryogenesis. Thus, the normalization of karyotype with ring chromosome is possible in vivo as well.

In iPSCs, cases of “compensatory” UPD of chromosomes 17 and 13 with duplication of intact chromosome were described by Bershteyn et al*.*^21^, who assumed that mitotic inheritance of ring chromosomes in iPSCs is very unstable and that ring chromosomes are incompatible with pluripotency. An analysis of iPSCs from a patient with ring chromosome 14 also showed the appearance of cells with a normal karyotype in one of the three cell lines analysed^46^. Our results suggest that spontaneous correction of the karyotype with ring chromosomes in iPSCs is not a universal phenomenon but rather a rare event. Of the four ring chromosomes studied, we observed this correction only for r(8). It is interesting that no karyotype normalization occurred in any of our four iPSC lines with r(13) given that Bershteyn et al*.*^21^ found rescued karyotypes in a total of seven out of nine iPSC clones from two patients with r(13).

**Figure 6:**
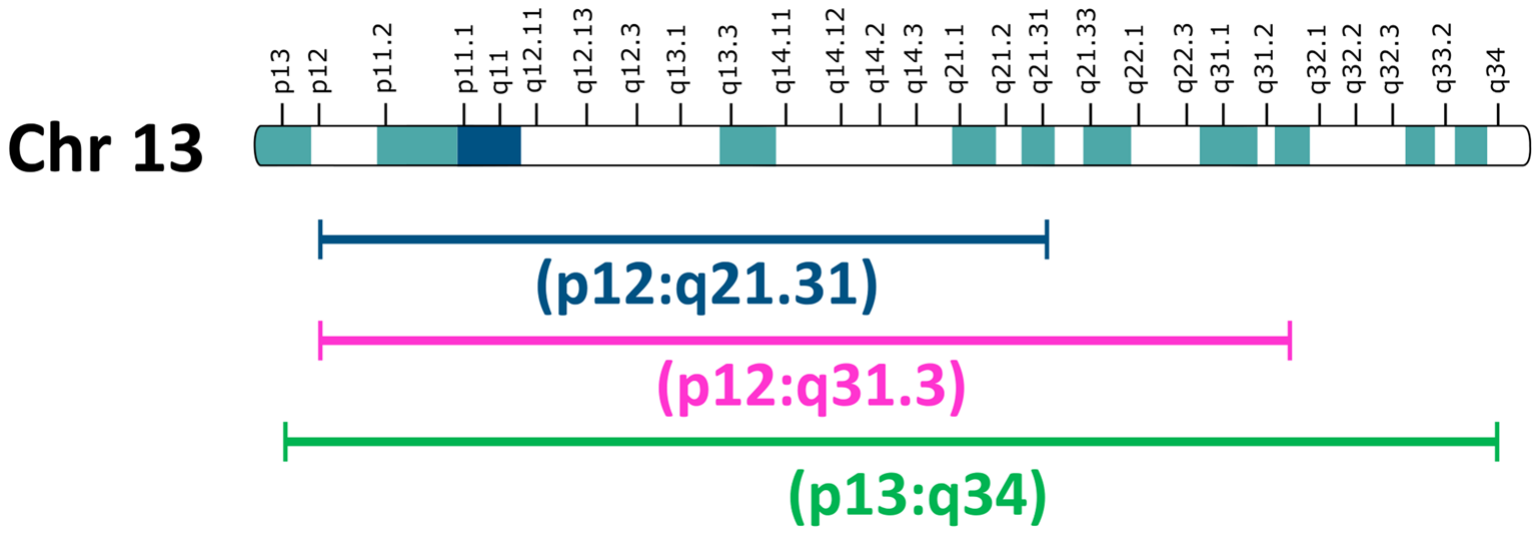
Schematic of chromosome 13 showing an approximate comparison of the length of r(13). The green line represents our patient, and the red and blue lines represent Bershteyn’s^21^ patients (Inkscape (v.0.91) (https://inkscape.org).

Can reprogramming to iPSCs trigger UPD emergence? Compensatory UPD is a two-step process with ring loss followed by duplication of the wild-type chromosome by non-disjunction^40,47^. Correction through reprogramming proper is possible in iPSCs if (i) the frequency of anaphase lagging and ring loss is relatively high; (ii) cells with the corresponding monosomy are less viable, so selective pressure is stronger; and (iii) duplication of the wild-type homologue is relatively frequent. More experimental information is required to explain this phenomenon, as molecular evidence of the ability of iPSCs to spontaneous karyotype correction is not currently available. Speculated causes of the differences in karyotype correction ability for different rings include the following:

(i) *The specific gene content of the chromosom*e. Ring chromosomes 8 and 17, both of which are chromosomes with cell-autonomous correction, include genes that contribute to accelerated iPSC proliferation. Trisomies of these chromosomes in iPSC lines of different origins are recurrent events^28,29,48^. The fact that Bershteyn et al*.*^21^ did not find r(17) in iPSC metaphases may be associated with the localization of the antiapoptotic *BIRC5* gene, which provides a proliferative advantage, in the 17q25 region^48,49^ such that ring instability or monosomy 17 may compromise the ability of pluripotent cells to divide more strongly than for other rings (r(13), r(18), and r(22)). Ring chromosome 18, which appeared in the ESC subline hESM01r18, was also quite stable^50^.
(ii) *The size of the chromosome.* A longer chromosome is assumed to be associated with a greater likelihood of SCE and subsequent problems with mitotic disjunction. In our study, the smallest r(22) (Table 1), was the most stable during long-term culture of iPSCs. However, r(13) in our study was physically larger than r(13) in Bershteyn’s study^21^ (Fig. 6), for which normalization of the karyotype via UPD was observed in most of the cell lines, which contradicts this assumption. Perhaps the preservation of a larger number of genes on r(13) in our case enabled relatively better survival of cells with a ring chromosome.
(iii) *Individual differences*. Individual genetic background differences are known to be leading factors in the variability of gene expression in pluripotent stem cells^51^, and their ability to karyotype correction^52^.
(iv) *The reprogramming method and culture conditions.* There is much evidence that the stability of the iPSC genome may be affected by the reprogramming method^53,54^ and culture conditions^55–57^. In our study, we used mitomycin-inactivated mouse embryonic fibroblasts as feeder and performed mechanical passaging—the combination most favourable for maintenance of genetic stability^56^.

Untangling spontaneous karyotype correction mechanisms is very important, as it paves the way for potentially attractive therapeutic approach for large-scale chromosomal aberrations^21,22^.

In general, we found some similarities in the “behaviour” of a particular ring chromosome between terminally differentiated cell (fibroblasts) and pluripotent cells, but we observed greater mitotic instability in most iPSCs (except those with r(22)) than in fibroblasts, especially at the sub-microscopic level. We hypothesize that the difference in ring chromosome inheritance in iPSCs from differentiated somatic cells is due to:

(i) The reprogramming process and the accelerated cell cycle in iPSCs cause replication stress and increase the frequency of double-stranded breaks (DSB)^55,58,59^.
(ii) DSB repair in iPSCs, unlike in somatic cells, occurs predominantly by the homologous recombination (HR) pathway^60,61^.
(iii) When HR occurs, the Holliday junctions between chromatids are formed, resulting in increased SCE formation in the S phase of the iPSC cell cycle.
(iv) As a result, the frequencies of dicentric and interlocked rings increases, followed by anaphase lagging, non-disjunction or fragmentation, resulting in monosomic cells or cells with multiple rings, anaphase bridges and micronuclei. Various ring derivatives of different sizes or fragmented rings may be produced in subsequent cell divisions (Fig. 5).

Surprisingly, we found, for the first time, that pluripotency is compatible with a wide range of derivative karyotypes. Two cell lines with r(13) consisted predominantly of cells with loss of most of the genetic content of the chromosome and retained pluripotency.

The results of this study emphasize that when dealing with ring chromosomes, it is important to reprogram fibroblasts to iPSCs as early as possible, since genomic alterations can occur during cell culture. The translocation variant t(7:8) arose in fibroblasts from a patient with r(8) before P6 and became predominant due to the proliferative advantage. This finding is even more relevant for iPSCs because the accumulation of cells with aberrant karyotypes is accelerated in pluripotent cells. Early passages of iPSCs are optimal for creating cellular models of chromosomal diseases.

In this study, characterization of the mitotic instability of ring chromosomes in pluripotent cells revealed complex mitotic behaviour at both the chromosomal and microstructural levels. Since iPSCs model early stages of embryonic development and are similar to ESCs, research on the generation of additional chromosomal rearrangements in iPSCs can help elucidate the origins of ring chromosome mosaicism in different cell types that are not available for analysis in patients in vivo but are clinically important.

## Methods

Cells from patients with de novo constitutional ring chromosomes 8, 13, 18, and 22 were used in the study. All experimental protocols and procedures were in accordance with the Declaration of Helsinki for Medical Research involving Human Subjects and the study was approved by the Scientific Ethics Committee of the Institute of Medical Genetics of the Tomsk National Research Medical Center (TNRMC) (protocol number 2017/106). Written informed consent was obtained from all parents for themselves and their children.

Medical records were collected. The phenotypic and developmental features of patients with r(8) and r(22) have been published earlier^23,31^. The patient characteristics are summarized in Table 1. Blood samples were obtained from each affected individual and his/her parents for chromosome preparations and DNA extraction. Skin biopsies from the patients were used for primary fibroblast culture establishment. FISH was used to visualize the ring chromosomes in lymphocytes and, in some cases, in cultured skin fibroblasts from the probands. Fibroblasts from early passages (2–3) were reprogrammed to iPSCs. The GTG karyotypes of cultured fibroblasts and iPSCs were examined at different passages, and aCGH analyses were performed for lymphocytes, fibroblasts and all iPSC lines.

*Karyotype analysis* was performed using conventional GTG banding techniques according to standard cytogenetic protocols based on the International System for Human Cytogenetic Nomenclature (2016). Twenty metaphase plates were counted for lymphocytes; for fibroblasts and iPSCs, we read at least 25–50 metaphases for each sample (at 450-band resolution) using Axioskop imaging microscope (Zeiss, Germany) with automatic karyotyping system Metafer (MetaSystems, Germany).

*FISH* was performed for lymphocytes and, in some cases, for cultured skin fibroblasts from the probands following a standard protocol. Images were captured using an Axio Imager Z2 (Zeiss, Germany) with ISIS software (MetaSystems, Germany). *E. coli* clones carrying plasmids with inserted centromere-specific alpha-satellite DNA sequences for the chromosomes studied as well as BAC clones were kindly provided by Professor M. Rocchi (Resources for Molecular Cytogenetics, Institute of Genetics, Bari, Italy). The probes for the unique genes *TBC1D22A*, *TUSC3*, and *UNC5D* were generated using a long-range PCR kit (BioLabMix, Russia) as described previously^62^. The probes were labelled with TAMRA-dUTP or Fluorescein-dUTP (BioSan, Russia) using nick translation.

*aCGH analysis* was performed on DNA from peripheral blood, fibroblasts, and iPSCs using a SurePrint G3 Human CGH Microarray Kit (8 × 60K) or a SurePrint G3 Human CGH + SNP Microarray Kit (4 × 180K) (Agilent Technologies USA), according to the manufacturer’s recommendations. Labelling and hybridization of the examined and reference DNA (#5190-3796, Human Reference DNA, Agilent Technologies) were performed using enzymatic labelling and hybridization protocols (v. 7.5, Agilent Technologies). Array images were acquired with an Agilent SureScan Microarray Scanner (Agilent Technologies). Data analysis was performed using CytoGenomics software (v. 3.0.6.6 or v. 5.0.2.5.) (Agilent Technologies).

*Cell culture* was performed on human fibroblasts that were derived from skin biopsies of patients with DMEM/F12 (Gibco) growth medium supplemented with 10% foetal bovine serum (FBS) (HyClone), 1% Pen-Strep (Gibco), 1% MEM non-essential amino acid (NEAA) solution, 1% MEM vitamin solution, and 2 mM l-glutamine (all from Sigma) at 37 °C in 5% CO_2_. For passaging, fibroblasts were washed with a Versene solution and incubated with 0.25% trypsin at 37 °C. The split ratio was routinely 1:2.

Established iPSC lines were cultured on mitomycin C-treated CD-1 mouse embryonic fibroblast feeder cells in iPSC medium [DMEM/F12 medium (Gibco) supplemented with 20% KnockOut Serum Replacement, 1% GlutaMAX, 1% MEM NEAA solution, 1% Pen-Strep (all from Gibco), 0.1 mM β-mercaptoethanol, and 10 ng/ml bFGF (Invitrogen)]. All cell cultures were maintained at 37 °C in 5% CO_2_. iPSCs were expanded mechanically.

*Generation of iPSCs from patient fibroblasts* was performed. The conditions under which the iPSC lines were obtained and the characteristics of the lines have been described in detail previously^24,25,29,31^. Skin fibroblasts from a patient with r(13) and r(22) were reprogrammed into iPSCs using modified LeGO-G2 lentiviral vectors (Addgene, #25917) with eGFP substituted by the human reprogramming transcription factors OCT4, SOX2, C-MYC, and KLF4 [LeGO vectors were kindly provided by Dr Sergei L. Kiselev (Moscow, Russia)].The lentiviruses were produced in the Phoenix cell line using Lipofectamine 3000 (Invitrogen) according to the manufacturer’s recommendations. Fibroblasts plated on the previous day were transduced with viruses containing the four reprogramming transcription factors for two days (on the second day, the C-MYC lentivirus was omitted). From day 6 to 16 the culture medium was changed daily with addition of 1 mM valproic acid (Sigma). On day 5, the transduced cells were seeded onto 10-cm culture dishes (2 × 10^3^ cells per cm^2^) containing mitomycin C-treated CD-1 mouse embryonic fibroblast feeder cells in iPSC medium. On day 18, colonies with iPSC morphology were picked up and expanded.

iPSC lines from fibroblasts with r(8) and r(18) were obtained using episomal vectors^63^. Fibroblasts were electroporated with a Neon Transfection System, and the transfection efficiency was 23.5%. Cells (0.5 × 10^6^) were electroporated with plasmids carrying *OCT3/4* (pCE-hOCT3/4, #41813), *SOX2* and *KLF4* (pCE-hSK, #41814), *L-MYC* and *LIN28* (pCE-hUL, #41855), a variant of the *p53* gene (pCE-mp53DD, #41856), EBNA1 (pCXB-EBNA1, #41857), and eGFP to evaluate the transfection efficiency (pCE-GFP, #41858). All plasmids were used in equal proportions so that the total amount of DNA was 6 μg.

The established iPSC lines had human ESC-like morphology; nuclear expression of the pluripotency markers OCT4, NANOG and SOX2; and expression of the surface markers SSEA4, TRA-1-60 and TRA-1-81, as detected by immunofluorescence staining. RT-PCR analysis confirmed that iPSC lines expressed pluripotency-associated markers, including *OCT4*, *NANOG,* and *SOX2*. In vitro spontaneous differentiation, followed RT-PCR, proved the ability of the iPSCs to differentiate into cells of the three germ layers. The iPSC lines iPSC-r(8)-2, iPSC-r(13)-1, iPSC-r(18)-3, iPSC-r(22)-1, and iPSC-r(22)-2 have been registered in the database *hpscreg*, and the corresponding names are listed in Supplementary Table S1.

*Immunocytochemistry* was conducted on iPSCs that were fixed in 3% paraformaldehyde for 20 min at 4 °C before being permeabilized in the presence of 0.1% Triton X-100 for 5 min. Blocking of nonspecific sites was achieved by incubation with 5% FBS for 20 min at room temperature. The cells were incubated with primary antibodies overnight at 4 °C and with secondary antibodies for 1 h at room temperature, antibodies listed in Supplementary Table S3. Nuclei were stained with VECTASHIELD with DAPI (Vector Laboratories), and immunofluorescence was visualized under an Axio Imager fluorescence microscope (Zeiss) with ISIS software (MetaSystems).

*In vitro differentiation* was performed using previously published protocol^64^. For embryoid body formation iPSCs were cultured in iPSC medium without bFGF on the agarose-covered plates. Cell aggregates were growing during 16 days, the media was change every other day.

*RT-PCR* was performed with HP-Taq DNA polymerase. Total RNA was isolated by TRI Reagent (Sigma) according to manufacturer's recommendations. The RNA was treated with DNase I, cDNA was obtained by RevertAid RT kit (both from Thermo Fisher Scientific). We analyzed the expression of endodermal (*HNF-3B, AFP, SOX17*), ectodermal (*SOX1*, *MAP2*, *PAX6*) and mesodermal (*TBXT (BRACHYURY), FLK1*, *MSX1*) genes in the obtained embryoid body cells, and expression of pluripotency-associated markers *OCT4*, *NANOG,* and *SOX2,* in iPSC*. GAPDH* was used as an endogenous control gene. The list of primers is shown in Supplementary Table S3.

*STR analysis* was performed to authenticate fibroblast-derived iPSC lines with their parental fibroblasts lines by Gordiz (http://gordiz.ru/). The loci analyzed were: D1S1656, D2S441, D3S1358, D5S818, D7S820, D8S1179, D10S1248, D12S391, D13S317, D16S539, D18S51, D21S11, D22S1045, AMEL, CSF1PO, FGA, SE33, TH01, TPOX, and vWA. The STR profiles of iPSC lines completely matched with that of the parental fibroblast cells.

The molecular cytogenetic and molecular genetic studies were performed at the Core Medical Genomics Facility of the Tomsk National Research Medical Center (NRMC) of the Russian Academy of Sciences using the resources of the biocollection “Biobank of the population of Northern Eurasia” of the Research Institute of Medical Genetics, Tomsk NRMC (http://medgenetics.ru/Biobank/). iPSC line derivation and differentiation was performed at the Collective Center of ICG SB RAS “Collection of Pluripotent Human and Mammalian Cell Cultures for Biological and Biomedical Research” (http://ckp.icgen.ru/cells/; http://www.biores.cytogen.ru/brc_cells/collections/ICG_SB_RAS_CELL).

## Supplementary information

Supplementary information.

## Data Availability

Data on iPSC lines are available from *hpscreg* database (https://hpscreg.eu/) with accession numbers listed in Supplementary Table S1. All other relevant data supporting the key findings of this study are available within the article and its Supplementary Information files. STR and SNP data are available from the corresponding author upon reasonable request.
