# Supplementary material for: Complex biology of constitutional ring chromosomes structure and (in)stability revealed by somatic cell reprogramming
Source: Sci Rep. 2021 Feb 22;11:4325. doi: 10.1038/s41598-021-83399-3 (PMC7900208; doi:10.1038/s41598-021-83399-3)
Supplement: Supplementary file 1 — Supplementary information. [file 41598_2021_83399_MOESM1_ESM.pdf]

Complex biology of constitutional ring chromosomes structure and (in)stability revealed by somatic cell reprogramming

Nikitina T.V., Kashevarova A.A., Gridina M.M., Lopatkina M.E., Khabarova A.A., Yakovleva Yu.S., Menzorov A.G., Minina Yu.A., Pristyazhnyuk I.E., Vasilyev S.A., Fedotov D.A., Serov O.L., Lebedev I.N.

Supplementary information

Supplementary Table S1. Correspondence of the cell line names used in this and others articles/database

Ring chromosome	Name in this article	*hpscreg* database and *Stem Cell Research* articles	Laboratory name
8	iPSC-r(8)-1		iTAF11-1
	iPSC-r(8)-2	ICGi025-A[25]	iTAF11-4
	iPSC-r(8)-3		iTAF11-5
	iPSC-r(8)-4		iTAF11-9
	iPSC-r(8)-5		iTAF11-11
	iPSC-r(8)-6		iTAF11-12
13	iPSC-r(13)-1	IMGTi003-A[26]	iTAF6-6
	iPSC-r(13)-2		iTAF6-23
	iPSC-r(13)-3		iTAF6-25
	iPSC-r(13)-4		iTAF6-36
18	iPSC-r(18)-1		iTAF12-3
	iPSC-r(18)-2		iTAF12-6
	iPSC-r(18)-3	ICGi024-A[30]	iTAF12-19
22	iPSC-r(22)-1	IMGTi001-A[32]	iTAF5-29
	iPSC-r(22)-2	IMGTi001-B[32]	iTAF5-32

Supplementary Table S2. Antibodies used for immunocytochemistry analysis of pluripotency

	**Antibody**	**Dilution**	**Company Cat # and RRID**
Primary antibodies	Rabbit anti-NANOG	1:100	Abcam Cat# 21624, RRID: AB_446437
	Rabbit anti-OCT4	1:200	Abcam Cat# 19857, RRID: AB_445175
	Mouse anti-SSEA4	1:600	Abcam Cat# 16287, RRID:AB_778073
	Mouse anti-TRA-1-60	1:600	Abcam Cat# 16288, RRID:AB_778563
	Mouse anti-TRA-1-81	1:600	Abcam Cat# 16289, RRID:AB_2165986
	Mouse anti -SOX2	1:400	RSE National center for Biotechnology, Astana, Cat# NCB 1601
Secondary antibodies	Alexa Fluor 546 anti-Mouse IgG	1:500	Thermo Fisher Scientific Cat# A-11060, RRID:AB_2534107
	Alexa Fluor 546 Goat Anti- Rabbit IgG	1:500	Life technologies Cat# A-11010, RRID:AB_143156
	Alexa Fluor 488 Goat Anti-Mouse IgG	1:500	Life technologies Cat# A-11029, RRID:AB_138404

Supplementary Table S3. Primers used for analyses of iPSC on pluripotency and differentiation capacity

	**Target**	**Forward/Reverse primer (5′-3′)**
Pluripotency markers	*OCT4*	CTGGGTTGATCCTCGGACCT / CACAGAACTCATACGGCGGG
	NANOG	AAAGAATCTTCACCTATGCC / GAAGGAAGAGGAGAGACAGT
	SOX2	GCATCGCAGCTTGGATACAC / GCTTCAGCTCCGTCTCCAT
House-keeping gene	*GAPDH*	GTGGACCTGACCTGCCGTCT / GGAGGAGTGGGTGTCGCTGT
Differentiation markers	*AFP*	AAATGCGTTTCTCGTTGCTT / GCCACAGGCCAATAGTTTGT
	TBXT (*BRACHYURY*)	AATTGGTCCAGCCTTGGAAT / CGTTGCTCACAGACCACA
	SOX1	CACAACTCGGAGATCAGCAA / GGTACTTGTAATCCGGGTGC
	PAX6	GTCCATCTTTGCTTGGGAAA / TAGCCAGGTTGCGAAGAACT
	MAP2	CAGGTGGCGGACGTGTGAAAATTGAG AGTG / CACGCTGGATCTGCCTGGGGACTGTG
	MSX1	CGAGAGGACCCCGTGGATGCAGAG / GGCGGCCATCTTCAGCTTCTCCAG
	SOX17	CTCTGCCTCCTCCACGAA / CAGAATCCAGACCTGCACAA
	FLK1	TGATCGGAAATGACACTGGA / CACGACTCCATGTTGGTCAC
	HNF-3B	GGAGCGGTGAAGATGGAA / TACGTGTTCATGCCGTTCAT

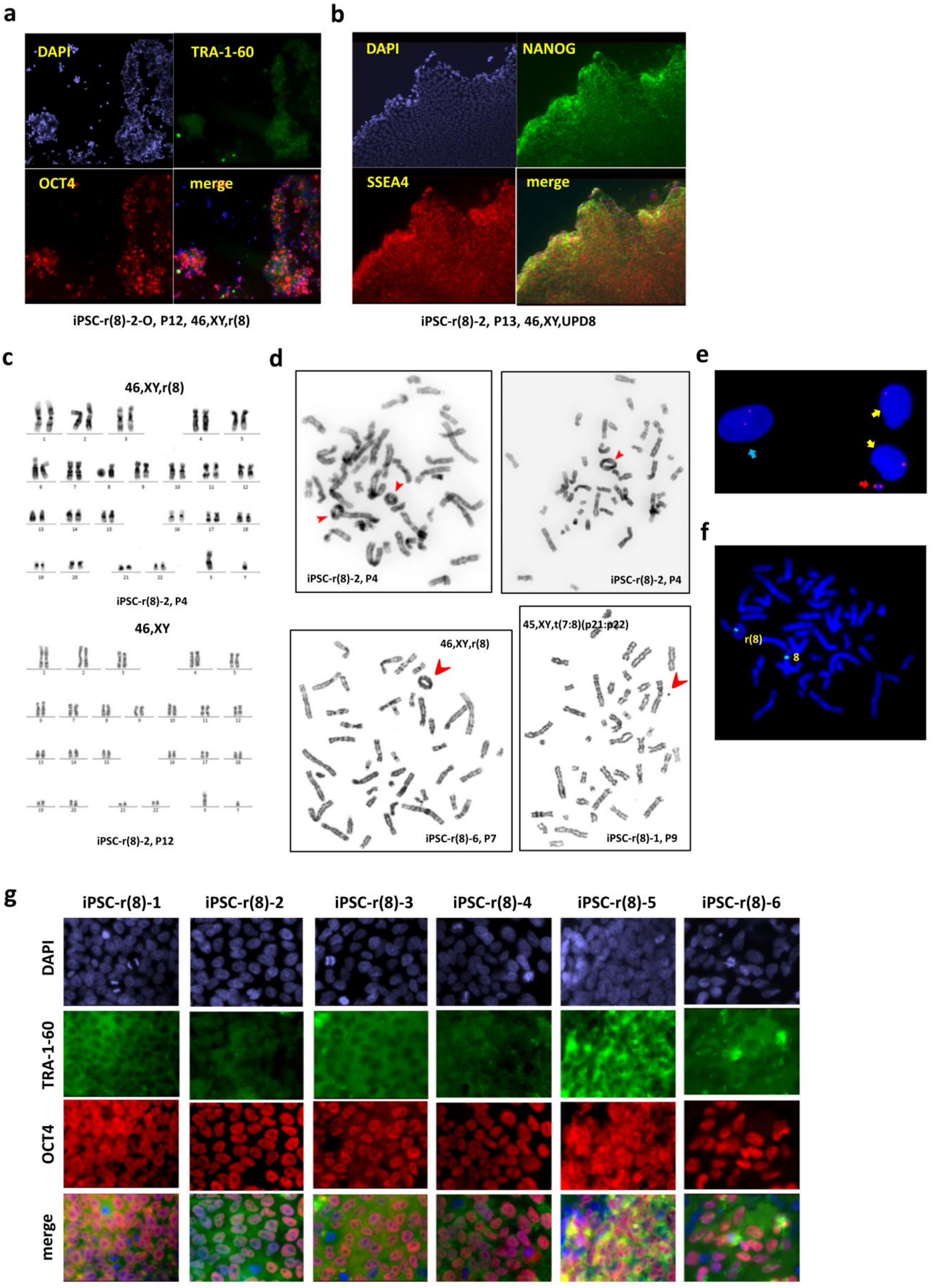

Supplementary Fig. S1. (a) Immunocytochemistry with antibodies against the pluripotency markers OCT4 and TRA-1-60 for subclone iPSC-r(8)-2-O at P12 showed degradation of morphology of colonies and decreases in the expression of pluripotency markers (ISIS software (v.5.5), MetaSystems (https://metasystems-international.com/en/products/isis/)). **(b)**

Immunocytochemistry with antibodies against the pluripotency markers NANOG and SSEA4 for clone iPSC-r(8)-2 at P13 with high expression of pluripotency markers and ESC-like morphology of colonies (ISIS software (v.5.5), MetaSystems (https://metasystems-international.com/en/products/isis/)). **(c)** Karyotypes 46,XY,r(8) in iPSC-r(8)-2 line at P4 and 46,XY at P12. **(d)** Examples of iPSC metaphase spread with two rings, with one ring and with translocation t(7:8). **(e)** Interphase FISH analysis with the centromeric probe D8Z2 (red) showing disomy 8 (green arrow), monosomy 8 (yellow arrows) and centromere-positive micronucleus (red arrow) (ISIS software (v.5.5), MetaSystems (https://metasystems-international.com/en/products/isis/)). **(f)** FISH analysis with the centromeric probe D8Z2 (green) showing ring and normal chromosome 8 (ISIS software (v.5.5), MetaSystems (https://metasystems-international.com/en/products/isis/)). **(g)** Immunocytochemistry with antibodies against the pluripotency markers OCT4 and TRA-1-60 for six iPSC lines studied (ISIS software (v.5.5), MetaSystems (https://metasystems-international.com/en/products/isis/)).

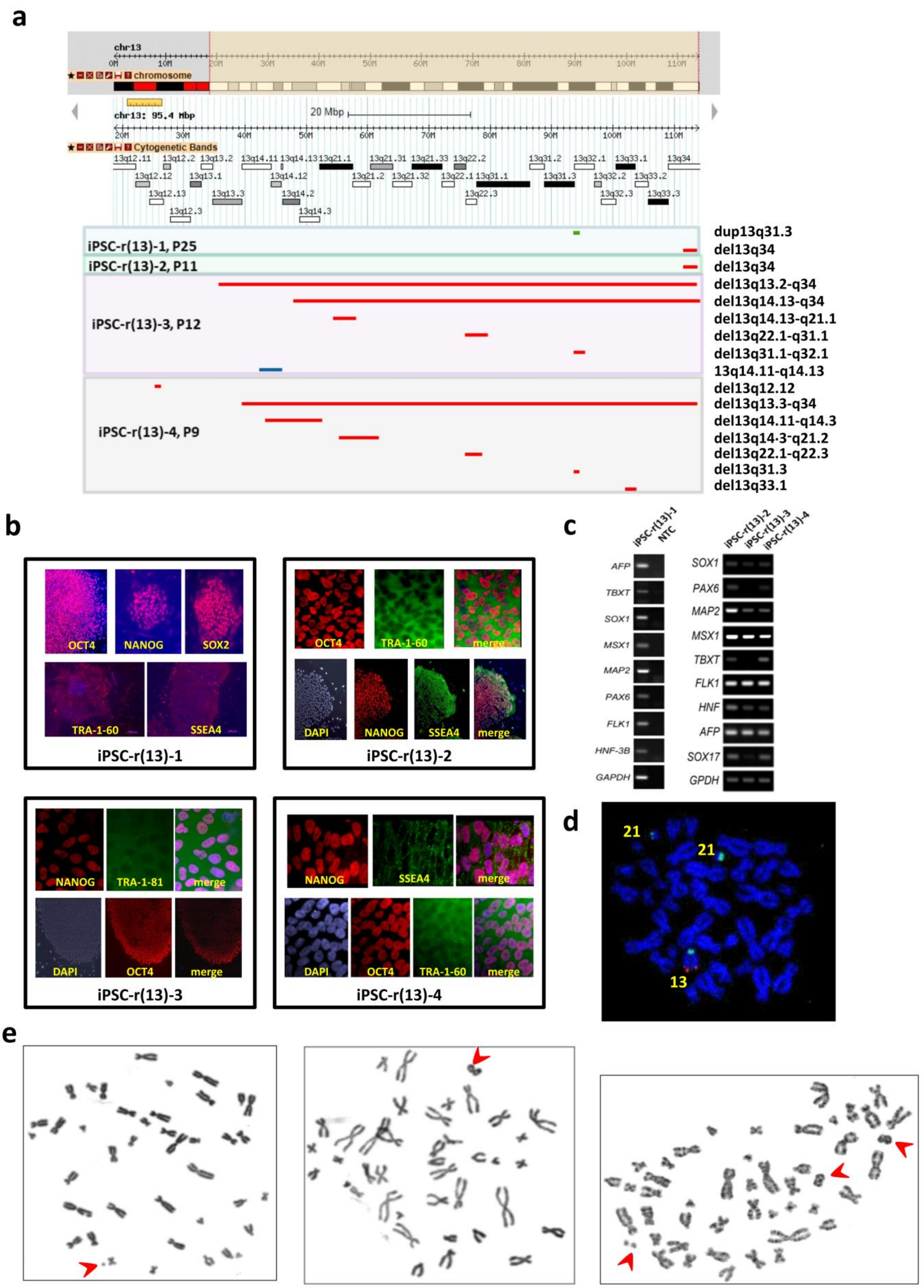

Supplementary Fig. S2. (a) Map of microdeletions on chromosome 13 in four iPSC clones, showing microstructural stability of lines iPSC-r(13)-1 and iPSC-r(13)-2, and instability of lines iPSC-r(13)-3 and iPSC-r(13)-4. From top to bottom: common view and cytogenetic

bands of the q-arm of chromosome 13 (taken from Database of Genomic Variants, available online: http://projects.tcag.ca/variation, accessed on 06 January 2021), CNVs on chromosome 13 in four iPSC clones (our own data). **(b)** Expression of pluripotency markers in four iPSC lines with r(13) (ISIS software (v.5.5), MetaSystems (https://metasystems-international.com/en/products/isis/)). **(c)** RT-PCR analysis of the of the ability to form three germ layer derivatives in the embryoid bodies in four iPSC lines. **(d)** FISH analysis showing monosomy 13 (green: centromeric 13/21 probe D13Z1; red: probe RP11-569D9 on the distal part of the long arm of chromosome 13 (13q34)) (ISIS software (v.5.5), MetaSystems (https://metasystems-international.com/en/products/isis/)). **(e)** Examples of metaphase spread (from left to right): with marker chromosome (derivate 13); with r(13); with two r(13) and two marker chromosomes.

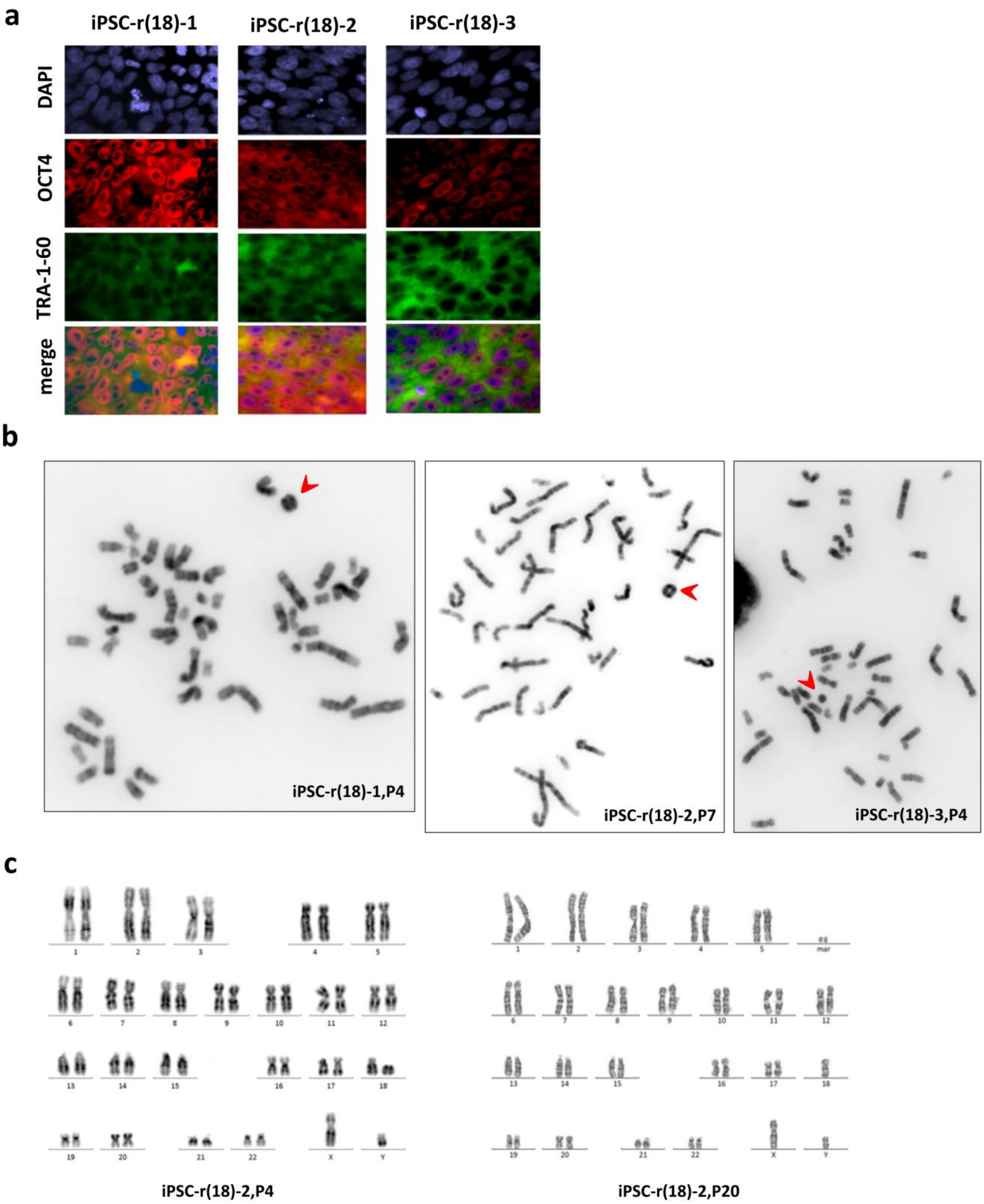

Supplementary Fig. S3. (a) Immunocytochemistry with antibodies against the pluripotency markers OCT4 and TRA-1-60 in three iPSC lines with r(18) (ISIS software (v.5.5), MetaSystems (https://metasystems-international.com/en/products/isis/)). **(b)** Examples of metaphase spread with dicentric ring chromosome 18 (left) and monocentric r(18). **(c)** Karyotyps with r(18) (left) and with two marker chromosomes (right).

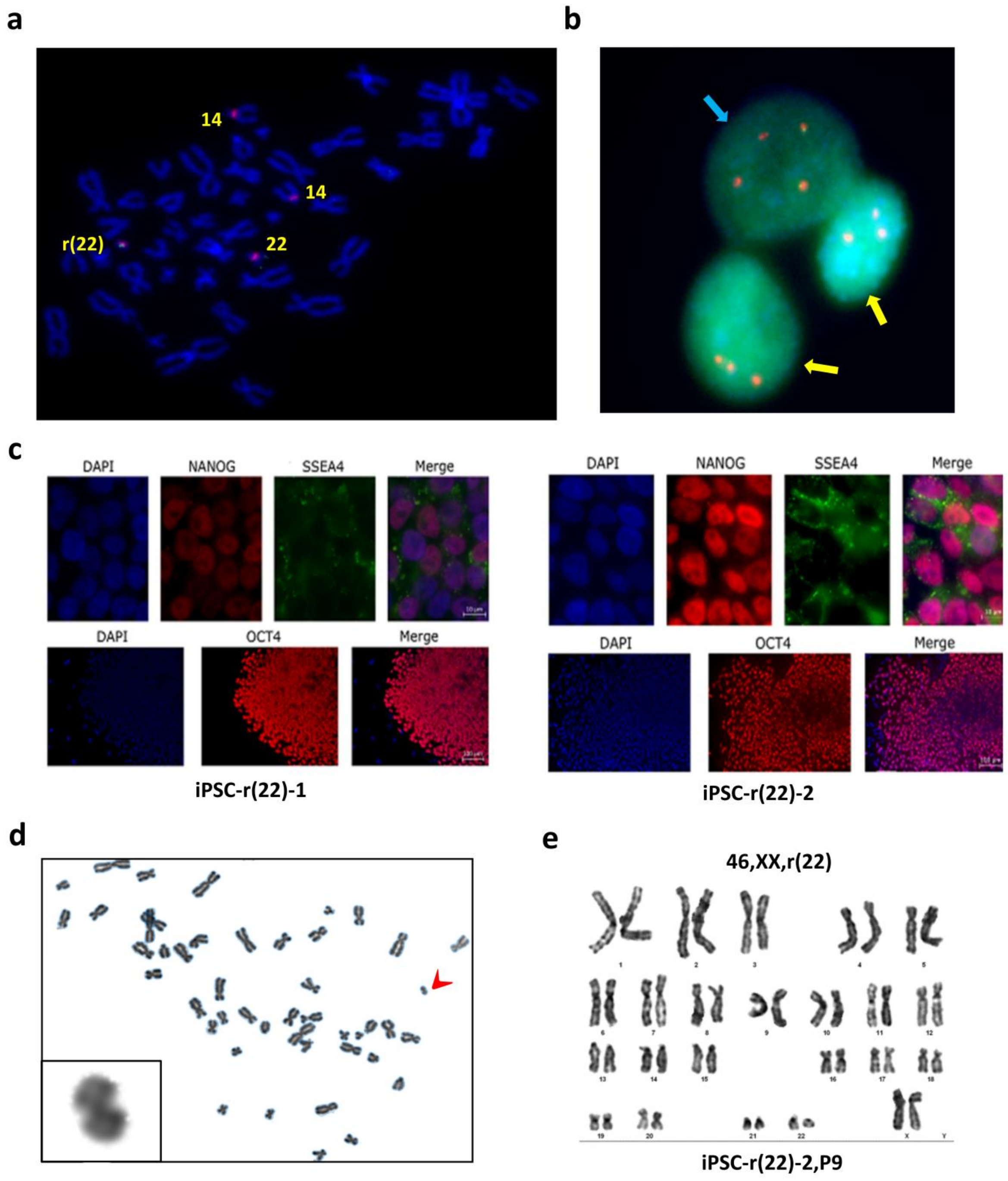

Supplementary Fig. S4. (a) FISH analysis showing normal and ring chromosomes 22 (red: centromeric probe 14/22, green: unique gene *TBC1D22A* (22q13.33)) (ISIS software (v.5.5), MetaSystems, (https://metasystems-international.com/en/products/isis/)). **(b)** Interphase FISH with the centromeric probe 14/22, blue arrow – disomy 22, yellow arrows – monosomy 22 (ISIS software (v.5.5), MetaSystems (https://metasystems-international.com/en/products/isis/)). **(c)** Immunocytochemistry with antibodies against the pluripotency markers NANOG and SSEA4 (×60) и OCT4 (×10) for lines iPSC-r(22)-1 and

iPSC-r(22)-2 with high expression of pluripotency markers and ESC-like morphology of colonies (ISIS software (v.5.5), MetaSystems (https://metasystems-international.com/en/products/isis/)). **(d)** Example of metaphase spread with r(22). **(e)** Karyotype 46,XX,r(22) in iPSC-r(22)-2 line.
